# Mitochondrial microRNAs: A Putative Role in Tissue Regeneration

**DOI:** 10.3390/biology9120486

**Published:** 2020-12-21

**Authors:** Sílvia C. Rodrigues, Renato M. S. Cardoso, Filipe V. Duarte

**Affiliations:** 1 Exogenus Therapeutics, 3060-197 Cantanhede, Portugal; silviacouto25@gmail.com; 2 Doctoral Programme in Experimental Biology and Biomedicine (PDBEB), Institute for Interdisciplinary Research (IIIUC), University of Coimbra, 3004-504 Coimbra, Portugal; 3 CNC—Center for Neuroscience and Cell Biology, University of Coimbra, 3004-504 Coimbra, Portugal; 4 LaserLeap Technologies, 3025-307 Coimbra, Portugal; renatocardoso83@gmail.com

**Keywords:** microRNA, mitochondria, mitomiRs, tissue regeneration

## Abstract

**Simple Summary:**

Distinct tissue engineering strategies are currently being developed. Cell-based therapies, implantation of synthetic scaffolds or combination of a scaffold with seeded cells are being used according to the biologic premises. Mitochondria play a central role in cell life cycle due to their preponderant role in energy production. Recent data suggest that small non-coding RNAs encoded by mitochondria DNA such as microRNAs regulate a plethora of factors and consequently signaling pathways, crucial in disease pathogenesis and also putatively in regenerative processes. Unveiling mitochondrial microRNAs biological function and their targets will propel the development of innovative therapeutic and diagnostic tools.

**Abstract:**

The most famous role of mitochondria is to generate ATP through oxidative phosphorylation, a metabolic pathway that involves a chain of four protein complexes (the electron transport chain, ETC) that generates a proton-motive force that in turn drives the ATP synthesis by the Complex V (ATP synthase). An impressive number of more than 1000 mitochondrial proteins have been discovered. Since mitochondrial proteins have a dual genetic origin, it is predicted that ~99% of these proteins are nuclear-encoded and are synthesized in the cytoplasmatic compartment, being further imported through mitochondrial membrane transporters. The lasting 1% of mitochondrial proteins are encoded by the mitochondrial genome and synthesized by the mitochondrial ribosome (mitoribosome). As a result, an appropriate regulation of mitochondrial protein synthesis is absolutely required to achieve and maintain normal mitochondrial function. Regarding miRNAs in mitochondria, it is well-recognized nowadays that several cellular mechanisms involving mitochondria are regulated by many genetic players that originate from either nuclear- or mitochondrial-encoded small noncoding RNAs (sncRNAs). Growing evidence collected from whole genome and transcriptome sequencing highlight the role of distinct members of this class, from short interfering RNAs (siRNAs) to miRNAs and long noncoding RNAs (lncRNAs). Some of the mechanisms that have been shown to be modulated are the expression of mitochondrial proteins itself, as well as the more complex coordination of mitochondrial structure and dynamics with its function. We devote particular attention to the role of mitochondrial miRNAs and to their role in the modulation of several molecular processes that could ultimately contribute to tissue regeneration accomplishment.

## 1. Regenerative Biology

Regeneration is the ability of animals to restore, either partially or entirely, tissues or organs that were damaged due to trauma [1]. This regenerative capacity strongly varies along the animal kingdom and, while some species can regenerate the whole body from just small fragments (hydra and planaria), there are other species, such as mammals, that have a poor regenerative capacity, and a profound damage generally results in scarring [2,3]. Since the 18th century, evolutionary biologists have been trying to disclose the mechanisms underlying the tissue regeneration in animals with a higher regenerative capacity than humans, such as salamanders [4] and zebrafish [5,6]. This diversity in the regenerative capacity across animal species is attributed to different tissue repair mechanisms that can be classified in two categories: morphallaxis and epimorphosis [7]. While the first is common in lower species, the latter is usually observed in higher order species being attributed to the regeneration of the damaged tissue through a local cell proliferation mechanism (blastema) which does not affect the remaining organism [8]. The epimorphic regeneration has been widely studied in salamanders, zebrafish and other species and it involves different stages that are initiated after an injury and culminated with the perfect regeneration of the tissue or organ. In its initial stages, the epimorphic regeneration is very similar to a wound-healing mechanism. After injury, there is the hemostasis phase followed by a re-epithelization process in the damage epidermis (migration of the keratinocytes to the injured cite) [8]. As the re-epithelization is complete, the regeneration stage starts with the formation of a mass, composed of heterogeneous undifferentiated cell populations (blastema), and those cells have the capacity to differentiate in specific cells that are required to complete the repair of the tissue [9]. There are some similarities between epimorphic regeneration and scar formation: they are both triggered by a hemostatic process and there is a re-epithelization process to cover the wounded area, with the formation of blastema being the main difference between both regenerative processes. It is known that in most mammals, the regenerative process stalls in the early stages of wound healing and then moves towards a fibrotic repair and scar formation [9]. Considering this, the ultimate goal of regenerative medicine is the stimulation of a blastema that would ultimately drive the healing process towards regeneration instead of scarring. Although the overall capacity of mammals, namely humans, is somewhat limited, their organs have differential self-renewal capacities and, while some organs continuously regenerate, like blood and skin, others can regenerate after injury, such as liver and bone, and others have a restricted regeneration capacity, like the heart and kidneys [10].

### Clinical Approaches

**Figure 1:**
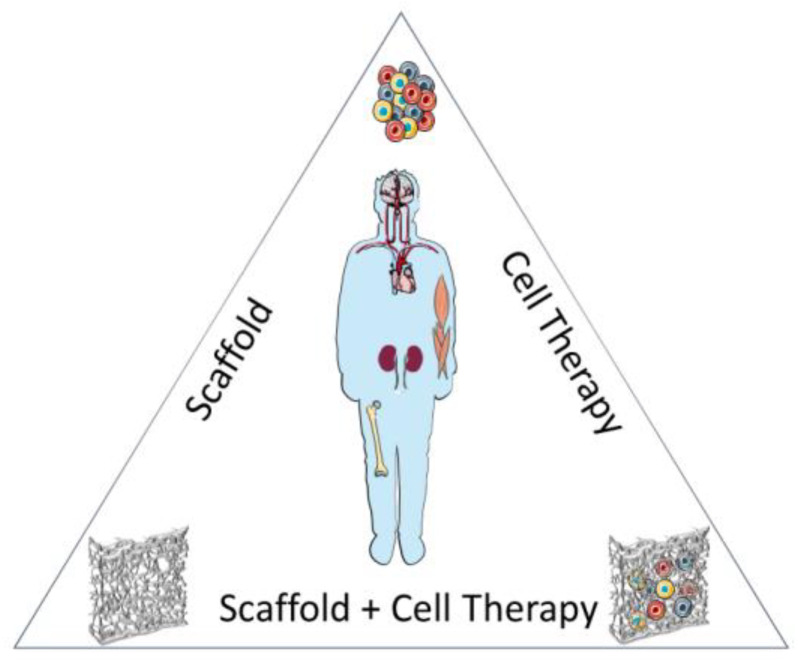
Depiction of the current clinical/therapeutic approaches to tissue regeneration: cell-based therapies, implantation of synthetic scaffolds to repair and combination of a scaffold with seeded cells.

Currently, there are three different approaches in regenerative medicine: (i) cell-based therapies, (ii) implantation of synthetic scaffolds to repair and (iii) combination of a scaffold with seeded cells [11], as depicted in Figure 1. Cell therapy consists in the injection at the injured site of either differentiated cells or undifferentiated stem cells to reconstitute the damaged tissue. Stem cells, due to their ability to differentiate into several cell types, emerged as a fundamental tool in regenerative medicine. Initially, the therapeutic potential of stem cells was attributed to their capacity to differentiate in tissue-specific cells, therefore promoting tissue regeneration. Nowadays, it is known that stem cells also actively secrete a broad variety of biomolecules (cytokines, chemokines, growth factors) that either act on neighboring cells (paracrine effect) or on themselves (autocrine) to promote tissue repair.

In regenerative medicine, human stem cells can be classified into tissue-specific progenitor stem cells (TSPSCs), embryonic stem cells (ESCs), umbilical cord stem cells (UCSCs), mesenchymal stem cells (MSCs) and induced pluripotent stem cells (iPSCs) [12]. The ESCs are capable of differentiation into mesodermal, endodermal and ectodermal cell lineages, and that makes them outstanding tools for regenerative medicine [13]. According to the literature, the transplantation of hESCs to patients with spinal cord injury (SCI) improves sensation, body control and overall limb movement [14]. Moreover, ESCs-derived cardiovascular progenitor cells regenerate cardiac tissue, providing its mechanical repair [15,16]. ESCs-derived hepatocytes have been successfully accomplished and they are an important tool for drug screening and cell therapy [17]. The transplantation of ESCs-derived chondrocytes effectively heals knee joint defects [18]. Although ESCs have emerged as a promising tool in regenerative medicine, their use in research has been highly debated due to ethical concerns [19]. TSPSCs, in opposition to ESCs, give rise to specific types of cells; moreover, their number is limited, making collection and in vitro expansion extremely difficult. When activated by a signal delivery, TSPSCs start to proliferate and migrate to the injury site, where they differentiate and acquire a specific phenotype contributing to tissue repair and renewal. In a clinic environment, a positive correlation between long-term metabolic success upon transplantation of pancreatic progenitor cells was demonstrated, proving that despite their limitations, the cells have an important regenerative capacity (induction of expandable tissue-specific progenitor cells from human). The MSCs have emerged as a versatile and widely used therapeutic tool in regenerative medicine. They are found in several in vivo locations such as bone marrow, skin, pancreas, heart, brain, lung and others [20,21,22], and can be easily expanded without losing their multipotency capacity. MSCs, when exposed to specific factors, can undergo a trilineage differentiation: osteogenesis, adipogenesis and chondrogenesis [23]; however, the differentiation towards other cell types has also been reported in the literature [24]. The umbilical cord blood provides an immune-compatible source of stem cells that is easily and safely collected and stored by UCB banks [25]. Moreover, UCBSCs have also been considered as an alternative for ESCs due to their content in multipotent stem cells, able to differentiate in several cell types [26]. Currently, the regenerative capacity of UCBSCs has been described for several diseases that include neuronal injury [27], cardiac infarction [28] and bone regeneration [29].

First developed by Takahashi and Yamanaka in 2006, iPSCs can differentiate into all cell types, and they can be obtained from adult somatic cells by the induction of the four transcription factors [30,31]. These cells have a high proliferative capacity and have been used to derive skin cells [32,33], epithelial stem cells [34] and fibroblasts [35], among others. Additionally, IPSCs have been successfully generated from fibroblasts, isolated from patients with recessive dystrophic bullosa, which were further differentiated in keratinocytes to reconstruct human skin structure [36]. Although there are several pre-clinical evidences that IPSCs may be a promising therapeutic approach in regeneration, their clinical application has been delayed due to their potential tumorigenicity [37].

The natural tissues consist in cells and growth factors that are embedded in an extracellular matrix (ECM). The ECM is a complex fibrous nanostructured matrix that gives both structural and functional support to cells. It controls the mechanical and biological properties of the tissue, playing a key role in gene expression and cell dynamics (survival, death proliferation, differentiation and migration) [38]. Synthetic or natural scaffolds have been studied in regeneration, they have a three-dimensional (3D) architecture and are designed to replicate the ECM mechanical and microenvironmental properties [39]. When associated with cells, the scaffolds should be tailored to provide support, allowing the cells to survive, differentiate and migrate, improving the efficacy of cell transplantation and regeneration [40]. Moreover, these materials can be designed to function as a reservoir, releasing the therapeutic bioactive molecules in a spatially controlled manner [41]. One of the major challenges regarding cell transplantation in regenerative medicine is their long-term survival, given that most of the injected cells die in a few hours either due to the lack of growth factors, insufficient support from the tissue (low vascularization) or the transplantation procedure (mechanical stress) [42]. The biomaterials may be tailored to address and overcome these challenges, significantly increasing the cell survival in transplantation. Material scaffolds can modulate and control the stem cell fate by providing specific environmental conditions (mechanical and biochemical) [43]. By changing the stiffness of several materials, MSCs increased the expression of markers corresponding to osteogenic, myogenic and neurogenic lineages [44,45]. Moreover, the biomaterial porosity and adhesion properties are crucial for cell engraftment [46]. Nowadays, biomaterials constitute an indispensable tool for regenerative medicine therapies, namely cell transplantation, they allow to improve cell survival, enabling the tissue regeneration and the clinical translation of cell therapies. Currently, 963 studies, either completed or in phases 3/4, use stem cells and address a wide array of diseases and pathological conditions (wound healing, infertility, bone regeneration, etc.). From those studies, only 9 involve the use of a scaffold or a matrix to support and deliver the cells, suggesting that the field of biomaterials still has a path to travel before its clinical reality (www.clinicaltrial.gov).

## 2. Mitochondria miRNA Biology

MicroRNAs (miRNAs) are endogenous small, single-stranded molecules of noncoding RNA (19–23 nucleotides) that represent a new level of control of gene expression. miRNAs act at the post-transcriptional level to modulate protein-coding genes, either by blocking the translation of messenger RNA (mRNA) or by actively encouraging its degradation, and it is well reported that each miRNA can target multiple genes [47]. Given that miRNAs are now postulated as master regulators and fine tuners of gene expression, these molecules might enclose relevant diagnostic, prognostic and therapeutic applications.

miRNAs are currently documented as relevant players in almost every biological process, from cell proliferation to differentiation, apoptosis and organogenesis [47,48]. The finding and the study of miRNAs has noticeably changed the classical understanding of gene expression and our comprehension of the biogenesis and function of miRNAs has markedly grown in recent years. Some associations between miRNA deregulation and human disease have been reported in different medical fields [49]. The research in this new ‘arena’ has exposed the enormous potential of miRNAs as tools for diagnostics or therapeutics. More specifically, several studies have already explored the modulation of mitochondrial DNA genetics by miRNAs [50]. Mitochondria, besides chloroplasts in plants, are the only organelles that possess a separate genome, the so-called mitochondrial DNA (mtDNA). Mutations in this DNA molecule have been shown to be involved in an assortment of both physiological (e.g., heat production, reactive oxygen species (ROS) production, apoptosis, cellular differentiation and aging) and pathological traits, including neurodegenerative diseases, diabetes, metabolic syndrome and cancers [51,52]. Additionally, there is a plethora of crosstalk signals that allow the communication between the nuclear and mitochondrial genomes. This mechanism of regulation is vital for the activity of the whole cellular machinery, basically by modulating mitochondrial biogenesis and metabolism [53] through reciprocal mitochondrial-to-nucleus communication [54,55].

The most famous role of mitochondria is to generate ATP through oxidative phosphorylation fueled by a chain of four protein complexes, the electron transport chain (ETC). An impressive number of more than 1000 mitochondrial proteins have been discovered [56]. Mitochondrial proteins can have a distinct genetic origin. It is predicted that ~99% of these proteins are nuclear-encoded and are synthesized in the cytoplasmatic compartment, being further imported through mitochondrial membrane transporters. The lasting 1% of mitochondrial proteins are encoded by the mitochondrial genome and the mitochondrial ribosome (mitoribosome) is responsible for the translation of these mRNAs. Moreover, the mitochondrion has its own protein synthesis machinery. As a result, an appropriate regulation of mitochondrial protein synthesis is absolutely required to achieve and maintain normal mitochondrial function.

**Figure 2:**
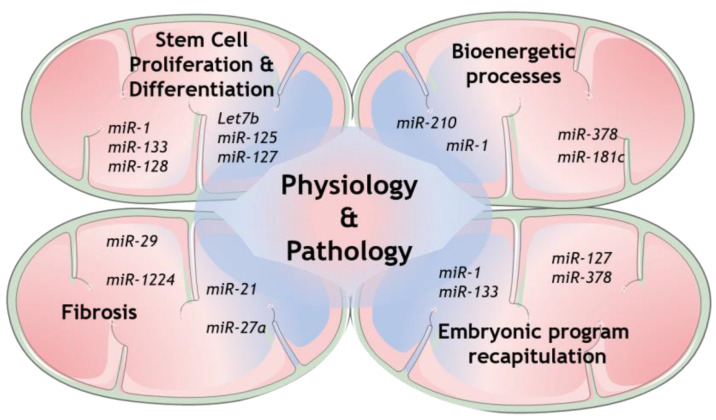
Modulation of physiologic and/or pathologic mechanisms by mitochondrial miRNAs. Several mechanisms that contribute to physiopathology require some biological functions that are modulated by different mitochondrial microRNAs.

Regarding miRNAs in mitochondria, it is well-recognized nowadays that several cellular mechanisms involving mitochondria are regulated by many genetic players that originate from either nuclear- or mitochondrial-encoded small noncoding RNAs (sncRNAs). Growing evidence collected from whole genome and transcriptome sequencing highlight the role of distinct members of this class, from short interfering RNAs (siRNAs) to miRNAs and long noncoding RNAs (lncRNAs) [57]. Some of the mechanisms that have been shown to be modulated are the expression of mitochondrial proteins itself, as well as the more complex coordination of mitochondrial structure and dynamics with its function [58,59,60]. The mechanisms involved in mitochondrial miRNAs transport are now being increasingly exposed and therefore more and more light is being shed upon mitochondrial miRNAs’ targets to determine their role in this unmapped cellular context [57,61]. Indeed, several studies have already disclosed the presence of miRNAs in mitochondria [59]. However, the mechanism by which the nuclear-encoded miRNAs are imported into mitochondria is still not fully established (see Reference [61] for a follow-up). On the other hand, numerous mitomiRs may be originated straight inside mitochondria, from mitochondrial genome-derived mRNA. Undeniably, mitomiRs typically act by regulating gene expression inside mitochondria at the post-transcriptional level [62]. Further than that, some of the mitomiRs may also target nuclear-encoded mRNAs localized on the mitochondrial surface [57]. Taken all together, these discoveries clearly reveal the significant role of mitomiRs in regulating mitochondrial gene expression and mitochondrial functions in both physiology and pathology [63], as summarized in Figure 2.

## 3. Mitochondrial miRNAs—Potential Contribution for Regeneration

Tissue repair and regeneration depends on miRNA regulation as these small molecular silencers play a fundamental part in post-transcriptional gene silencing. Multiple key biological processes important for regeneration such as cell growth and proliferation, differentiation and apoptosis, as well as mitochondrial function, are tightly controlled by them [64,65]. Regulation of miRNA expression levels is crucial as small changes in basal conditions are sequentially propagated and amplified throughout different biological pathways, ultimately leading to changes in cell phenotypes [66]. Therefore, a plethora of aspects in tissue regeneration could potentially be controlled by the manipulation of miRNAs.

Currently, different evidence shows that both nuclear-encoded miRNAs imported to mitochondria and mitochondrial genome-derived miRNA, both defined as mitomiRs, may influence mitochondrial dynamics, a fundamental process for tissue homeostasis (Table 1) [67,68]. In fact, regulation of how and how much energy cells require strongly impacts stem cells’ fate, controlling the ability of stem cells to decide when to exit from their quiescent state.

**Figure 3:**
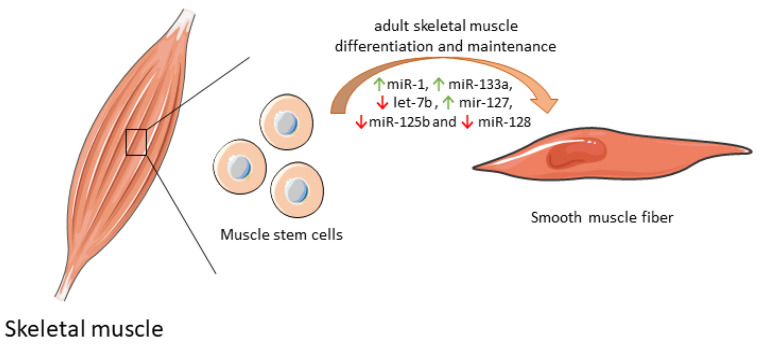
Differential expression of mitochondrial miRNAs impact adult skeletal muscle cell differentiation and maintenance. By modulating energy supply through mitochondria, these mitomiRs are able to promote the full differentiation of muscle fibers. Increased expression of the mitochondrial gene is needed to promote an adequate mitochondrial performance and to allow more rapid responses to physiological needs, namely tissue regeneration.

Some mitochondrial miRNAs were shown to modulate the differentiation of muscle stem cell into functional muscle cells by regulating mitochondria biogenesis (Table 1). By itself, miR-1 expression increases protein synthesis, and ATP production essential for optimal cell differentiation [69]. However, when miR-1 is simultaneously silenced with miR-133a in adult muscle stem cells in vitro, the expression of some mitochondrial genes was reduced, and atypical mitochondria were formed. Furthermore, a parallel in vivo experiment in a *miR-1/133a* double-knockout mouse model confirmed compromised muscle performance, as a consequence of mitochondrial dysfunction and impaired metabolic maturation [70]. let-7b, which has a dual role both on mitochondrial dynamics as well as on differentiation and maintenance of adult muscle cells [71], inhibited skeletal muscle growth by blocking cell proliferation and promoted cell cycle arrest and myofibroblast proliferation via the IGF-2 signaling pathway in vitro [72]. miR-127 was shown to mediate muscle stem cell differentiation through direct targeting sphingosine-1-phosphate receptor 3 (S1P3). Forced expression of miR-127 powered cell differentiation into skeletal cells both in vitro and in vivo. In a miR-127 transgenic mouse suffering Duchenne muscular dystrophy, administration of this biomolecule induced skeletal muscle regeneration and improved muscular dystrophy through stem cell differentiation [73]. Overexpression of other mitochondrial miRNAs, such as miR-125b and miR-128, was also correlated with inhibition of muscle stem cell differentiation and consequently impaired muscle regeneration [74]. Of note, miR-125b contributes to this inhibition through *insulin-like growth factor 2 (IGF-2)* targeting [75], while miR-128 suppresses specificity protein-1 (Sp1) [76]. In contrast, inhibition of miR-128 overexpresses Sp1 protein levels, abolishing proliferation and increasing differentiation [76]. The differential expression of mitomiRs that impact adult skeletal muscle cell differentiation and maintenance are depicted in Figure 3.

**Figure 4:**
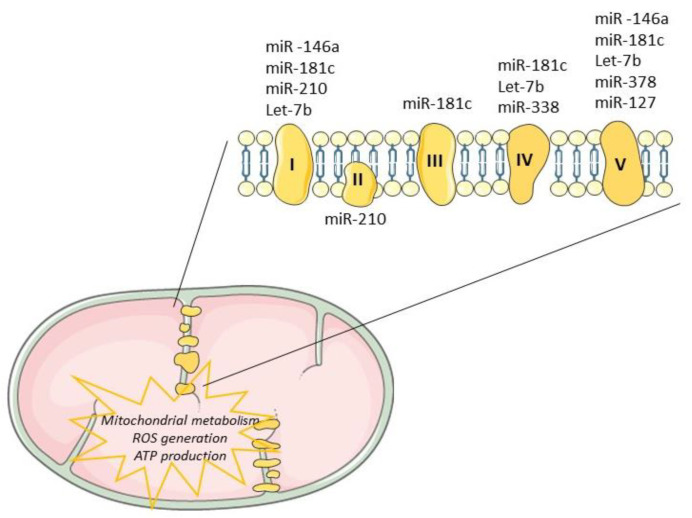
Mitochondrial miRNAs modulate metabolic processes. Mitochondrial miRNAs have been shown to regulate the mitochondrial electron transport chain by targeting multiple proteins. By impacting mitochondrial metabolism, ROS generation and ATP production, mitomiRs are important contributors for tissue regeneration.

Tissue regeneration after injury is a complex and a highly metabolically demanding process [77]. Thus, regulation of the mitochondrial genome by mitomiRs may impact the expression of key components driving ATP synthesis, consequently influencing regenerative mechanisms (Table 1). Mitochondrial miR-181c, miR-1, miR-338 and miR-210 have been shown to target multiple proteins that modulate the mitochondrial electron transport chain [57,78], as depicted in Figure 4. The impact of miR-181c was demonstrated both in vitro [79] and in vivo [80] and mechanistically described by binding to 3’UTR mitochondrial *cytochrome c oxidase subunit (COX)-1*, which affects the function of mitochondrial electron transport chain complex IV. While forced overexpression of miR-181c altered mitochondrial metabolism and ROS generation, contributing to heart failure, its inhibition may be enough to balance mitochondrial bioenergetics, potentiating cardiac remodeling or at least controlling cardiac damages. Recently, Banavath et al. showed that the loss of miR-181c, through *MICU1* upregulation, a specific promoter of Sp1, may protect the heart from injury [81]. Mitochondrial miR-338-5p in neural cell culture systems was demonstrated to modulate the expression of *COX-IV* and subunits of the ATP synthase complex [82,83]. When anti-miR-338 was transfected into axons, the metabolic oxygen consumption was increased by about 50% when compared with the nontargeting cells, potentiating cell survival. Furthermore, miR-378 was shown to have an important role in cardiac remodeling. This miRNA is highly expressed in diabetic cardiac mitochondria and it reduces ATP synthase activity by decreasing mitochondrial *ATP6* expression. This effect was further tested in vitro by overexpression of mitomiR-378 in HL-1 cells [84]. Consistently, in vivo delivery of miR-378-3p antagomir preserved ATP6 protein levels which balance the bioenergetic deficits, contributing to an adequate cardiac pump function [84].

Another way to envision tissue regeneration is by recapitulating, to some extent, the embryonic program that gave rise to the original tissue [85]. In this way, comprehending cellular and molecular mechanisms throughout embryonic development may help to pinpoint some important pathways to recapitulate in the regenerative process. As expected, mitomiRs have an important role in embryogenesis. Of note, early stages of cardiogenesis are predominantly supported by miR-1 and miR-133a, which sustain the differentiation of embryonic stem cells and precursors into cardiac-specific muscle lineage [86]. In fibroblasts and fetal hearts, low or absent levels of miR-378 were described, whereas high levels of this molecule were identified in postnatal injured hearts [87]. miR-378, when overexpressed in cardiomyocytes, enhanced apoptosis by direct targeting of IGF receptor 1 and reduced signaling in the Akt cascade. Curiously, inhibition of miR-378 protected cardiomyocytes against ROS and hypoxia reoxygenation-induced cell death [87]. miR-378, although being nuclear-encoded, controls mitochondrial metabolism as well as energy homeostasis [88].

Additionally, during human development, levels of miR-127-5p decreased throughout time. In fetal tissues like the liver and heart, their metabolic activity is mainly glycolytic, which is then converted in oxidative phosphorylation. miR-127-5p was suggested to possibly perform an important role in controlling the bioenergetic cell pattern by targeting *ATP5B*. Consistently, the overexpression of this molecule was able to decrease the amount of ATP5B protein by 50% in vitro, actively participating in the bioenergetic changes [89].

Aging leads to increased cellular senescence and decreases the functionality of tissue-specific stem and progenitor cells which, in turn, is linked to a limited cellular regenerative capacity. Identifying which molecular and cellular pathways can potentially decline the tissue homeostasis and regenerative capacity will promote the development of new therapeutic approaches. Eliminate senescent cells, rebalance the chronic oxidative stress and modulate impaired signaling and protein quality control are some examples that may alleviate tissue deterioration and restore the regenerative capacity [90]. Again, mitochondria do play a fundamental role in the previously listed mechanisms since its depletion inhibited cell senescence, while it promoted proinflammatory phenotype and maintained glycolysis as the cell bioenergetic mode [71]. Notably, several mitomiRs have been associated to both senescence and inflammatory processes by targeting the mitochondrial genome. miR-146a-5p is one of these molecules that by regulating the function of complex I and IV of the mitochondrial electron transport chain is able to control both inflammation and senescence [71]. Mir-146-5p has mitochondrial-encoded proteins such as ND1, ND2, ND4, ND5, ND6 and ATP8 as putative targets [71]. Since complex I determines ROS production in dysfunctional mitochondria, miR-146-5p is able to regulate this production upstream, inducing cell senescent phenotype. Although ROS are important in physiological processes such as proliferation and adaptation to hypoxia, their excess causes irreversible damages [91]. Therefore, by increasing ROS production, miR-146a-5p sustains a chronic proinflammatory and oxidative stress state by also targeting superoxide dismutase 2 (SOD2), an enzyme with antioxidant potential [92], and Bcl-2, a known protein that regulates mitochondrial dynamics, namely fusion and fission [93]. Additionally, oxidative stress induces permeability transition pore opening in mammalian mitochondria, which further contributes to irreversible mitochondria damage and loss of function [94]. Recently, miR-762 has also been discovered as a metabolic regulator. Its suppression attenuated the decrease in intracellular ATP levels, increased ROS production and diminished mitochondrial complex I enzyme activity. Specifically, in cardiomyocytes and ischemia/reperfusion injury, targeting miR-762 may be an interesting avenue to study to ameliorate myocardial infarction damage [95].

MitomiRs-19b, -20a, -17 and -106 are the most downregulated miRNAs in several human senescent cell and in vivo aging models [96]. Specifically, in endothelial cells, mitomiR-181a, -34a and -146a were shown to be overexpressed in senescent cells when compared with young ones. Besides targeting Bcl-2, these miRNAs induce permeability transition pore opening and stimulate caspase-1 and 3 and interfere with apoptosis susceptibility [97].

In turn, let-7b miRNA expression increases with aging. Hmga2 transcriptional regulator is a known target of this miRNA whose expression is high in fetal neural stem cells. A study demonstrated that age was correlated with reduced stem cell numbers and self-renewal throughout the central and peripheral nervous systems in Hmga2-deficient mice. This evidence suggests an important role of let-7b miRNA in the decline in neural stem cell function whose inhibition would potentiate the maintenance of stemness [98]. Also, miR-181a expression rises during the senescent process in human dermal fibroblast, with its overexpression being sufficient to induce cell senescence [99].

As mentioned before, mitomiRs have been described as novel players in both physiologic and pathologic conditions [100,101]. miR-1 is responsible for protecting heart structure and functions against hypertrophy, maintaining cardiovascular health. Thus, miR-1 is one of the crucial regulators of pathological cardiac hypertrophy by fine-tuning the translation of different molecules, including eukaryotic initiation factor 4E (EIF4E), Mef2a, Gata4 and histone deacetylase 6 (HDAC6) [102,103]. MiR-1 also reduces the cardiac hypertrophic response by negatively impacting calmodulin involved in calcium signaling [104] via directly targeting heart-specific fat binding protein 2 (FABP3). This protein intrinsically correlates with heart enlargement and hypertrophy in patients. An opposite relationship between the expression of miR-1 in myocardial tissue and FABP3 level in circulation was observed [105]. Other authors found that miR-1 suppresses *fibulin-2* expression which, by consequence, stops activation of TGF*β* signaling and extracellular matrix remodeling in hypertrophic heart [106,107]. In addition, miR-133, which is amply expressed in animal and human heart muscle tissues, blocks hyperthyroidism-induced cardiac hypertrophy by silencing the expression of type 1 angiotensin II receptor [108]. This molecule also reduces cardiac remodeling through target to Akt and its downstream signaling molecules, such as Cdc42, Rho-A and Nelf-A/WHSC2 [109]. miR-378 and miR-497 are antihypertrophic biomolecules. Studies demonstrated that they are able to block IGF receptor 1, growth factor receptor bound protein 2, kinase suppressor of Ras 1, Ras activity, PI3K-Akt pathway, Mapk1-MAPK signaling and the Raf1-MEK1-ERK1/2 pathway [110], and interfere with translation of Sirt4 [111]. Also, miR-212/132 family [112] and miR-23a [113] target Foxo3 transcription factor in cardiomyocytes, which alleviates the hypertrophic clues, while miR-29a-3p hinders ET-1-induced hypertrophic response in cardiomyocytes by directly targeting 3′ UTR of *NFATc4* [114].

In the same way, modulating the magnitude of a fibrotic response would potentiate regeneration by switching off cellular pathways that progressively will drive scarring and degeneration of the tissue. Likewise, several mitochondrial miRNAs exert their antifibrotic activity by modulating the deposition of components of extracellular matrix (Table 1) [115]. MiR-101, through targeting of TGF*β*RI and c-Fos, decreases extracellular matrix proteins’ production and proliferation of fibroblasts. Induction of miR-101 expression improves cardiac performance decline caused by the fibrotic process [116,117]. MiR-122 controls TGF-*β*1 expression, an important factor for severe myocardial fibrosis found to be downregulated in patients [118]. By inhibiting furin, miR-24 can suppress differentiation and migration of cardiac fibroblasts via TGF*β*-smad2/3 signaling. Importantly, in vitro, heart function was partially recovered by injecting a synthetic precursor of miR-24 [119].

miR-29a/b/c was shown to promote an anti-fibrotic effect by directly decreasing the production of ECM components in different tissues such as heart, lung, kidney and liver. These miRNAs target different collagens, such as *FBN1, ELN1, MMP2* and *ITGB1* genes [120,121,122,123]. For example, in a lung fibrosis in vitro model driven by silica, upregulation of miR-29b promoted mesenchymal–epithelial transitions (EMT) and suppressed gene expression of extracellular matrix-related genes. Concomitantly, downregulation of miR-29b improved EMT by elevating the protein ratio of E-cadherin/vimentin and upregulated ECM-related genes like vimentin, alpha-smooth muscle actin, collagen type 1 and *Tgfb1* [124]. miR-1224-5p expression showed similar effects in a lung fibrosis model by targeting *BECN1* [125].

In contrast, miR-27a, via the TGF-β1/Smad3 signaling pathway, may contribute to fibrosis in streptozotocin-induced diabetic rats [126]. Overexpression of this mitomiR activates the TGF-β signaling pathway, a key pathway in fibrosis pathogenesis, increasing the production of connective tissue growth factor, fibronectin and collagen I [127]. miR-21 also has a profibrotic activity in lung, heart and kidney. The presence of this miRNA increases the production of TGF-b by two alternative pathways. In the canonical way, miR-21 targets Smad7 and suppresses Smad2/3 [128,129], otherwise, miR-21 acts via *Spry*1 on the ERK/MAPK pathway [130].

Collectively, these evidences suggest a possible role of mitomiRs in tissue regeneration-associated processes. Nevertheless, further studies may be conducted to confirm the direct relationship between these molecules and concrete injury scenarios.

## 4. Emerging Therapies in Regenerative Medicine

A novel therapy that has been attracting the attention of the scientific community is the use of small extracellular vesicles for regeneration of tissues. These biological nanoparticles (100–200 nm) are secreted by almost all cell types and contain several bioactive molecules in their interior (miRNAs, proteins, nucleic acids) that are protected by a lipid bilayer envelope [131]. These nanoparticles are up-taken by cells via endocytosis and release their bioactive cargo in the cytoplasm of the target cells [132]. There are several risks associated with cell therapies such as teratomas, immune rejection and poor engraftment, therefore the use of this cell-free therapy could represent an important asset in regenerative medicine. There are several examples of the regenerative potential of exosomes in different tissues in the literature. The regenerative capacity of exosomes isolated from multipotent mesenchymal stromal cells has been addressed in neurons by Xin et al. and they demonstrated that exosomes could successfully deliver miRNA-133b to neural cells and boost neurite outgrowth. Ibrahim et al. showed that the injection of cardiosphere-derived exosomes, in hearts of mice suffering from ischemia, promote cardiomyocyte proliferation and inhibit apoptosis [133]. Additionally, Nojima et al. demonstrated that hepatocyte-derived exosomes can induce hepatocytes’ proliferation in vitro and liver regeneration in vivo [134]. The impact of exosomes in other regenerative contexts, such as cutaneous wound healing, muscular regeneration and renal failure, has been also addressed, unveiling their outstanding potential as a cell-free regenerative tool for an easy clinical translation. The use of exomes in the clinic is the next step, given that currently, from 132 clinical studies, 4 have reached the final stages (phase 3–4) before commercialization (www.clinicaltrials.gov).

Nowadays, the gene therapy aims not only to correct a possible genetic abnormality in a patient, by introducing a completely functional gene, but also to widen and enhance the regenerative potential of cells by intentionally delivering new genetic material into the cell [135]. Regarding the genetic engineering of the cell, it is interesting to observe that with this methodology, one can potentiate the basal regenerative attributes of the cell and at the same time, cells can be used as a delivery vehicle. Moreover, the genetic modification of cells allows to overcome some drawbacks of the direct gene therapy, namely the potential risks of systemic immune reaction due to viral vectors and failure of the vector containment [136]. Genetic modifications of MSCs were already performed in different types of cancer, and both melanoma [137] and glioma [138] showed a reduction in tumor growth and an increased survival. For a regeneration purpose, MSCs have been modified to overexpress BMP-2, enhancing their bone regeneration capacity [139]. Bao et al. have genetically modified MSCs to express TNF-α receptor and treat cardiac dysfunction and inflammation after acute myocardial infarction [140]. The stem cell gene therapy has proven to be efficient in pre-clinical studies and is now being applied in patients with different regenerative contexts (diabetic foot ulcer, recessive dystrophic epidermolysis bullosa, peripheral nerve injury), being a promising tool for the regenerative medicine field.

Similarly, the therapeutic potential of miRNAs, namely mitochondrial ones, is promising as they have proven to be novel players in several diseases such as cancer, metabolic and neurodegenerative diseases [100,101]. However, firstly, the extent of mitomiRs’ regulatory effects must be assessed as well as delivery processes that allow mitomiRs to target mitochondria, minimizing off-target effects [141]. Several mitochondrial microRNAs would be attractive candidates or targets to improve tissue health, hampering their loss of function. The use of mimics or inhibitors could be an alternative and attractive tool to manipulate cellular and tissue miRNA levels.

## 5. Conclusions

Nowadays, mitomiRs are still an unexplored biomolecule niche full of potential in different areas, namely tissue regeneration. Their crucial role both in health and disease is being more and more dissected. Given that mitomiRs are implicated in regulating mitochondria, and that mitochondria are heavily implicated in tissue formation/regeneration, a better comprehension of the role of these molecules may pave the way for the emergence of innovative therapies in regenerative medicine.

## Figures and Tables

**Table 1:** Summary of mitomiRs function and its known targets.

miR	Target genes	Function	Reference
**miR-1**		↑ protein synthesis ↑ATP production	[69]
	*EIF4E, Mef2a, Gata4, HDAC6*	Regulation of cardiac hypertrophy	[102,103]
	*FABP3*	Heart enlargement and hypertrophy	[105]
	*Fibulin-2*	↓ TGFβ signaling ↓ extracellular matrix remodeling	[106]
**miR1/miR-133a**		↑ number of mitochondrial genesInfluence on mitochondria morphology	[70]
		↑ cardiac stem cell differentiation	[86]
**Let-7b**	*IGF-2*	↓cell proliferation ↑ cell cycle arrest ↑ myofibroblast proliferation	[72]
	*Hmga2*	↑ cell senescence	[98]
**miR-127**	*S1P3*	↑ cell differentiation	[73]
	*ATP5B*	Control of bioenergetic cell pattern	[89]
**miR-125b**	*IGF-2*	↓ stem cell differentiation	[75]
**miR-128**	*Sp1*	↓ stem cell differentiation	[76]
**miR-181c**	*COX1*	Altered mitochondrial metabolism and ROS generation	[79,80]
**miR-181a**		↑ cell senescence	[99]
**miR-338**		Modulate COX-IV and subunits of the ATP synthase complex	[82,83]
**miR-378**	*ATP6*	↓ ATP synthase activity	[84]
	*IGF receptor 1*	↑ apoptosis ↓ signaling in Akt cascade ↑ ROS generation	[87]
**miR-146-5p**	*ND1, ND2, ND4, ND5, ND6, ATP8, SOD3, Bcl-2*	↑ ROS generation ↑ cell senescence	[71,92,93]
**miR-762**	*ND2*	↓ intracellular ATP levels ↑ increased ROS production ↓ mitochondrial complex I enzyme activity	[95]
**miR-19b, miR-20a, miR-17, miR-106**	*Bcl-2*	↑ permeability transition pore opening ↑ caspase-1 and 3 ↑ apoptosis	[96,97]
**miR-133**	*type 1 angiotensin II receptor, Cdc42, Rho-A* and *Nelf-A/WHSC2*	↓ cardiac remodeling	[108,109]
**miR-212/132**	*Foxo3*	↑ cardiac remodeling	[112]
**miR-23a**	*Foxo3*	↑ cardiac remodeling	[113]
**miR-29a-3p**	*NFATc4*	↓ hypertrophic response	[114]
**miR-101**	*TGFβRI* and *c-Fos*	↓ Extracellular matrix production ↓ fibroblast proliferation	[116,117]
**miR-24**	*furin*	↓ differentiation and migration of cardiac fibroblasts via TGFβ-smad2/3	[120]
**miR-29 family**		Antifibrotic role	[120,121,122,123]
**miR-1224-3p**	*BECN1*	↑ EMT ↓ gene expression of extracellular matrix-related genes	[125]
**miR-21**	*Smad7*	Pro-fibrotic effect	[128]
	*Spry1*	Pro-fibrotic effect	[130]
**miR-27a**	*TGFb*	Pro-fibrotic effect	[126,127]
